# Non-bacterial thrombotic endocarditis in ovarian cancer: A systematic review

**DOI:** 10.1016/j.gore.2025.101751

**Published:** 2025-05-05

**Authors:** Mahalia Huba, Fahad Hussain, Saimanoj Guntaka, Awais Paracha, Pranav Sathe, Bhavya Parikh, Margot Noyelle, Umar Durrani, Himanshu Patel, Veena John

**Affiliations:** a Medical University of South Carolina, 268 Calhoun St, Charleston, SC 29425, USA; b Northwell Health, 300 Community Drive, Manhasset, NY 11030, USA; c Saint Louis University School of Medicine, 1402 S Grand Blvd, St. Louis, MO 63104, USA

**Keywords:** Non-Bacterial Thrombotic Endocarditis, Ovarian Cancer, Malignancy, Hypercoagulable State, Valvular Vegetations, Anticoagulation

## Abstract

**Background:**

Non-Bacterial Thrombotic Endocarditis (NBTE) is characterized by the formation of sterile vegetations on the heart valves, often due to an underlying hypercoagulable state in the setting of malignancy, inflammation, or autoimmune disease. NBTE is a rare condition with a reported incidence rate of between 0.9% and 1.6%. Among gynecologic malignancies, NBTE is predominantly observed in ovarian cancer. A comprehensive review of the literature was conducted to understand the association between NBTE and ovarian cancer.

**Methods:**

A search of PubMed/MEDLINE was conducted from database inception to January 2024 using terms related to “Non-Bacterial Thrombotic Endocarditis” and “Ovarian Cancer.” Studies were included if they involved patients diagnosed with both NBTE and ovarian cancer and excluded if they did not report confirmed NBTE and ovarian cancer. Risk of bias was assessed using Mixed Methods Analysis Testing.

**Results:**

The search identified 15 patients from 14 case reports, all female, with an average age of 50.6. The most common presenting diagnosis was CVA (53%). Ovarian cancer was diagnosed before NBTE 57% of the time. Most patients (67%) had stage III or IV cancer. Metastases predominantly affected lymph nodes (40%), liver (40%), and the other ovary (20%). Malignancy was treated with chemotherapy in 63% of cases. Surgical resection was performed in 40% of cases. One patient underwent radiation therapy. Mortality rate was 40% in patients treated with chemotherapy and 100% in patients not receiving chemotherapy. NBTE predominantly affected the mitral valve (52.4%), followed by the aortic valve at (33.3%), and the tricuspid valve (14.3%). 80% of patients experienced organ infarct. Infarcts predominantly affected the brain (27.0%), spleen (27.0%), lung (8.1%), and heart (8.1%). NBTE was treated with anticoagulation in 54% of cases. All-cause mortality at study publication was 75%.

**Conclusions:**

NBTE is an important, and often overlooked, cause of mortality in ovarian cancer. Clinicians should maintain high clinical suspicion for NBTE in patients with ovarian cancer presenting with thromboembolic phenomena.

**REGISTRATION:**

This review was registered with PROSPERO under the ID number: CRD42024501301.

## Highlights

• NBTE is a form of sterile endocarditis often associated with advanced malignancy and there is a lack of literature exploring its association with ovarian cancer.
• The most common presenting symptoms of NBTE in ovarian cancer were neurologic in nature.
• The majority of patients with NBTE-associated ovarian cancer were found to have late-stage (stage III or IV) malignancy.
• Valvular vegetations in NBTE associated with ovarian cancer predominantly affected left-sided heart valves.
• Chemotherapy was associated with a positive effect on mortality while the effects of anticoagulation and surgery were less clear.

## 1 Introduction

Nonbacterial Thrombotic Endocarditis (NBTE), also known as Marantic Endocarditis, is a condition characterized by non-infectious/sterile vegetations that occur on cardiac valves, consisting of fibrin and platelet aggregation (Lopez et al., 1987). NBTE is rare and has a reported incidence between 0.9 % to 1.6 % (Lopez et al., 1987). Prevalence has varied widely in clinical reports (from 0.3 % to 9.3 %), thought to be in part due to the variance in diagnostic methods (Lopez et al., 1987). The most frequently associated condition with NBTE is malignancy, with a reported incidence of 75–80 % (Llenas-García et al., 2007, Mazokopakis et al., 2010).

NBTE is associated with many conditions, with the most common being malignancy. Other etiologies include inflammatory, connective and autoimmune conditions such as systemic lupus erythematosus, antiphospholipid syndrome, rheumatic heart disease, tuberculosis, acquired immunodeficiency syndrome (AIDS), snake bites, and radiation therapy (Asopa et al., 2007).

The malignancies most frequently linked to NBTE include lung cancer, pancreatic cancer, gastric cancer, and adenocarcinomas of unknown primary origin (Rahouma et al., 2023). Interestingly, comparison between malignancies showed that adenocarcinomas (such as those of the lung, pancreas, and ovary) showed higher incidence rates, with the highest rates in mucin-secreting and pancreatic adenocarcinoma (el-Shami et al., 2007, Quintella et al., 1991, Borowski et al., 2005). Conversely, non-adenocarcinoma cancers (squamous cell, hematologic malignancies, sarcomas etc) account for a minority of NBTE cases and occur typically in the context of advanced disease (el-Shami et al., 2007, Quintella et al., 1991, Borowski et al., 2005). Although these associations were demonstrated across multiple autopsy series, the pathogenesis is still unclear.

Among gynecologic malignancies, NBTE is predominantly observed in cases of ovarian cancer (Delgado and Smith, 1975). Prevalence of NBTE in ovarian cancer is not well documented, and often, diagnoses of NBTE take place post-mortem (Orfanelli et al., 2016). A very recent study on cancer-induced NBTE found gynecologic malignancy to be the third-most common cancer associated with NBTE at 14.8 %, behind lung and pancreatic cancer, but further research looking at ovarian cancer and NBTE is very limited (Akiki et al., 2023). Data on prevalence of ovarian cancer are widely available, and it is the second-most common gynecologic malignancy in the US and other resource-rich countries (Siegel et al., 2024). The above statistics shed light on the dearth of data on NBTE prevalence in ovarian cancer despite an abundance of documentation and data available on ovarian cancer and its prevalence.

NBTE is rare and has a reported incidence between 0.9 % to 1.6 % in one study, and less than 1 % in another study (Lopez et al., 1987). Prevalence has varied widely in clinical reports (from 0.3 % to 9.3 %), thought to be in part due to the variance in diagnostic methods (Lopez et al., 1987). The most frequently associated condition with NBTE is malignancy, with a reported incidence of 75–80 %.(Llenas-García et al., 2007, Mazokopakis et al., 2010).

The pathophysiology of NBTE in cancer patients involves damage to valvular endothelial cells due to an underlying hypercoagulable and inflammatory state. The process involves macrophages and malignant cells interacting to release cytokines such as tumor necrosis factor and interleukins, leading to endothelial damage (Smeglin et al., 2008). This endothelial damage promotes local platelet aggregation, inflammatory mononuclear cell migration, and deposition of immune complexes, resulting in the formation of a thrombus intertwined with fibrin (Liu and Frishman, 2016). These vegetations are prone to dislodging and systemically embolizing because of a lack of inflammatory reaction at the site of the deposition (Aryana et al., 2006). Vegetations usually occur on the left side of the heart, most commonly affecting the mitral valve followed by the aortic valve, and occasionally involving both valves (Mazokopakis et al., 2010). While extremely uncommon, there are reported cases of all four valves being affected by NBTE (Laco et al., 2008).

Generally, patients with NBTE tend to be asymptomatic from a cardiac standpoint, and symptomatic presentation is usually due to systemic emboli and their sequelae rather than signs of heart failure or valve dysfunction. Valvular dysfunction is less commonly seen than symptomatic systemic emboli because the vegetations usually do not significantly alter valvular function (Mazokopakis et al., 2010). Common sites of systemic emboli include central nervous system, coronary arteries, kidneys, spleen, skin, and extremities with symptomatic presentation including cerebrovascular accident (CVA), myocardial infarction (MI), limb or digit ischemia, hematuria and flank pain among others.(el-Shami et al., 2007).

Diagnosis of NBTE is often challenging, as there are no validated laboratory tests or procedures to confirm the condition. The diagnosis is typically made clinically, after ruling out other, more common, causes of the patient’s presentation, such as infective endocarditis (Zmaili et al., 2022). This can be done through obtaining the appropriate work-up and using Duke’s Criteria to understand the risk of infective endocarditis. A work-up suspicious for NBTE could include negative blood cultures, low white blood cell counts, and low inflammatory blood marker levels of c-reactive protein, erythrocyte sedimentation rate (Liu and Frishman, 2016). Once NBTE is suspected, a thorough investigation for malignancy, systemic lupus erythematosus, antiphospholipid syndrome, and disseminated intravascular coagulation should be undertaken. Transthoracic echocardiography (TTE) and/or transesophageal echocardiography (TEE) are employed for visualizing vegetations. TTE is typically chosen initially for assessing heart function and volume (Liu and Frishman, 2016). TEE, offering higher sensitivity, is preferred for evaluating cardiac valve conditions, particularly detecting smaller (less than five millimeters) lesions or those on the tricuspid or pulmonic valve (Liu and Frishman, 2016). Cardiac magnetic resonance imaging can assist in distinguishing vegetations from neoplasms or thrombi (El ouazzani et al., 2020).

There has been no therapy that has been shown to reverse the valvular vegetations in NBTE, so current management of NBTE focuses on treatment of the underlying disease process (oftentimes malignancy) along with systemic anticoagulation. Per the American College of Chest Physicians’ guidelines on patients with cancer and NBTE, long-term anticoagulation with full-dose unfractionated heparin is considered essential and recommended (Salem et al., 2004). Many retrospective analyses favor unfractionated heparin over warfarin, direct thrombin inhibitors, or factor Xa inhibitors (Lopez et al., 1987). The American Heart Association also recommends use of heparin in conditions with intracardiac thrombi (Lopez et al., 1987). Specifically, data regarding efficacy of direct oral anticoagulants (DOACs), which include direct thrombin inhibitors and factor Xa inhibitors, have been limited to a few case reports, and lacks large-scale trials (Akiki et al., 2023). Indications for valvular surgery for NBTE follows the same guidelines as for infective endocarditis, and generally include symptomatic heart failure, vegetation size greater than 10 mm, symptomatic heart block or recurrent emboli despite appropriate medical management.

The prognosis of malignancy-associated NBTE is poor, with a high associated morbidity and mortality. A recent study on cancer-related NBTE found a mortality rate of 77.9 % during follow-up (Patrzalek et al., 2024). This poor prognosis likely stems from advanced stages of malignancy upon diagnosis of NBTE and complications from the underlying malignancy (Zmaili et al., 2021). No specific data are available on ovarian-cancer related NBTE and mortality rate.

Literature on NBTE in ovarian cancer is limited to case reports and series. While the existing data from these studies are valuable, it leaves us with a fragmented picture of patient presentation in NBTE associated with ovarian cancer, which we hypothesize might present differently than NBTE in general malignancy. In this review, we aim to examine trends in epidemiology, clinical presentation, diagnostic work-up and outcomes of NBTE in patients with ovarian cancer.

## 2 Methods

### 2.1 Literature search

This systematic review was registered with the Internal Prospective Register of Systematic Reviews (PROSPERO) under the ID: CRD42024501301. It was in accordance with Preferred Reporting Items for Systematic Reviews and Meta-Analyses (PRISMA) guidelines. A comprehensive literature search was conducted using PubMed/MEDLINE and searching for articles from the database inception to January 2024. The following primary search terms “NON-BACTERIAL THROMBOTIC ENDOCARDITIS” OR “NBTE” OR “MARANTIC ENDOCARDITIS” OR “STERILE ENDOCARDITIS”, OR “LIBMAN SACKS ENDOCARDITIS”, OR “CULTURE-NEGATIVE ENDOCARDITIS” were paired in all possible combinations with the terms “OVARIAN CANCER”, “EPITHELIAL CELL OVARIAN CANCER”, “GERM CELL OVARIAN CANCER”, and “CLEAR CELL OVARIAN CANCER”. Filters were applied to the search results to include only full-text articles, studies published in English, and studies including human subjects.

### 2.2 Study Selection

**Fig. 1:**
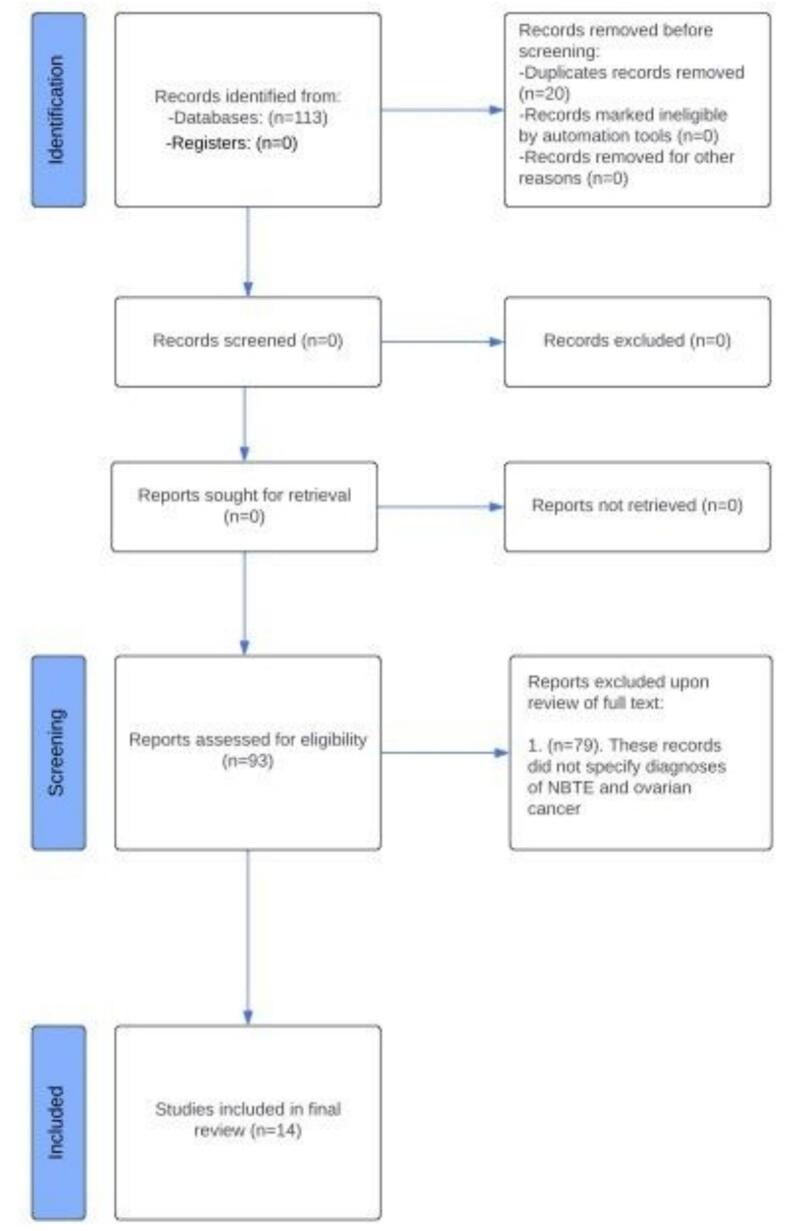
**Study Selection** All records were identified from the PUBMED/MEDLINE database and 113 records were initially identified. 20 of these records were duplicates and were removed by default. 93 records were assessed for eligibility and 79 were deemed ineligible as they did not specify confirmed diagnoses of NBTE and ovarian cancer. 14 total records met all criteria and were included in the study.

Two independent reviewers (F.H, P.S) assessed the eligibility of relevant papers using specific inclusion and exclusion criteria Fig. 1. The study inclusion criteria were broad and included all studies discussing NBTE and ovarian cancer. Filters were applied to include only full-text articles, articles published in English, and studies conducted on humans. Studies were excluded if they did not report confirmed NBTE and ovarian cancer or were duplicates of studies found in previous searches. They were also excluded if they were not pertaining to ovarian cancer or included various other forms of endocarditis (i.e., infective endocarditis) in the study. The studies were also removed if there was a presence of multiple cancers or cancers of unknown origin. Both reviewers independently assessed each record, and pertinent studies were individually screened for inclusion without any use of automation tools. We employed a consensus approach to evaluate all selected studies to avoid the risk of individual biases. Risk of bias was assessed for individual studies by two independent reviewers (U.D., F. H.) using the Mixed Methods Appraisal Tool.

### 2.3 Data Selection

Various outcomes of interest were collected related to the presentation, diagnosis, work-up, treatment, and mortality in patients with a diagnosis of NBTE associated with ovarian cancer. The data points collected from each pertinent study were the following: patient age, sex, past medical history, presenting symptoms, presenting diagnosis, presence or absence of organ infarcts, location of organ infarcts if present, heart valves affected by NBTE, presence or absence of metastases, the chronology of ovarian cancer or NBTE diagnosis, time to diagnosis of NBTE and ovarian cancer, patient physical exam findings, notable laboratory findings, blood culture findings, indications of hyper-coagulable state, echocardiogram findings, CT scan findings, whether empiric antibiotics were initiated, treatment (chemotherapy, radiation, anticoagulation, surgery) prescribed, overall patient outcomes, whether patient death was a direct result of NBTE or ovarian cancer, and other significant work-up. Results that were compatible with each data point were explored. One author (M.H.) collected the full complement of data from the selected studies and this data was checked for accuracy by multiple members of the team. Trends were analyzed and discussed in the following sections. In the instance of absent results for a data point, we reported percentages of the available data. These instances were noted in the results section. The results are also visually represented as two data tables, one representing characteristics of patient presentation Table 1 and the other representing characteristics of diagnostic work-up Table 2.

## 3 Results

### 3.1 Description of patients and neoplasms

Our review of the literature produced 14 individual case reports describing 15 patients with ovarian cancer and NBTE Table 1. The sample included 15 female patients with an average age of 50.6 years and a standard deviation ± 6.63. All patients had epithelial ovarian cancer and the subtypes described in this sample included serous adenocarcinoma of the ovary (n = 5) (Orfanelli et al., 2016, Sawai et al., 2018, Tanaka et al., 2009), clear cell carcinoma of the ovary (n = 1) (Lin et al., 2015), adenocarcinoma of the ovary (n = 1) (Aryana et al., 2006), endometrioid adenocarcinoma of the ovary (n = 1) (Numnum et al., 2006), and mucinous cystadenocarcinoma of the ovary (n = 1) (Kooiker et al., 1976). Six case reports did not specify the subtype of ovarian carcinoma. Borowski et al., 2005, Makhdumi et al., 2021, Yagi et al., 2014, Singh et al., 2007, Tadokoro et al., 2013.

**Table 1:** Patient characteristics.

Paper	Patient	Presenting Diagnosis	Organ Infarcts	Metastasis	Initial Diagnosis (NBTE or OC)	Valves Affected	Treatment (anticoagulation)	Cancer Treatment	Outcome	Cause of Death
**Makhdumi et al.**	48F	Aortic Regurgitation	None	None	Ovarian CA	Aortic	None	None	Not Reported	−
**Yagi et al.**	54F	DIC	Renal, Splenic, Brain	None	Ovarian CA	Aortic, Mitral	None	None	Dead	DIC
**Gilbert et al.**	58F	CVA	Kidney, Spleen, Brain	Right ovary, Focal migration to the left ovary, and Supracolic and Infracolic omentum	NBTE	Mitral	None	Carboplatin, Paclitaxel, and Surgery (tumor resection)	Alive	−
**Sawai et al.**	53F	Trousseau’s syndrome	Renal, Splenic, Bowel, Brain	Liver	Ovarian CA	Aortic	Unfractionated heparin and Rivaroxaban	Surgery (not specified)	Dead	Multiorgan failure
**Kooiker et al.**	51F	CVA	Brain, Heart, Spleen, Kidney	None	Same time during autopsy	Mitral	None	None	Dead	Cerebral Infarction
**Numnum et al.**	38F	Mild mitral valve prolapse	Splenic, Renal, Brain	None	NBTE	Aortic and Tricuspid	Warfarin	Paclitaxel and Carboplatin, Topotecan, Pegylated Doxorubicin, and Gemcitabine, Total abdominal hysterectomy, and Radiation	Alive	
**Singh et al.**	52F	CVA	Brain	None	NBTE	Mitral	Heparin	Carboplatin and Paclitaxel	Dead	Ovarian cancer
**Borowski et al.**	46F	CVA	Kidneys, Brain	Lymph nodes	NTBE	Aortic	Heparin	Hysterectomy and lymphadenectomy	Dead	Ovarian Cancer
**Mukai et al.**	40F	MI	Brain, Heart, Kidneys, Spleen	None	NTBE	Mitral and Aortic	None	None	Dead	DIC
**Lin et al.**	60F	CVA	Spleen and Kidneys	Lymph nodes	Ovarian CA	Mitral and Tricuspid	Ticlopidine, Warfarin, and Heparin	None	Dead	Sepsis
**Tadokoro et al.**	55F	CVA	None reported	Unknown	Ovarian CA	Mitral	Not stated	Not stated	Not stated	−
**Tadokoro et al.**	56F	CVA	None reported	Unknown	Ovarian CA	Mitral	Not stated	Not stated	Not stated	−
**Tanaka et al.**	46F	CVA	Brain, Kidney, Lungs, Spleen	None	NBTE	Mitral	Given but not specified	None	Dead	Decompensation leading to death
**Orfanelli et al.**	59F	MI	Pulmonary artery, Brain, Spleen, Kidney, Heart, and Left leg	Liver	Ovarian CA	Mitral, Tricuspid, and Aortic	Heparin	Carboplatin and Paclitaxel	Dead	DIC
**Aryana et al.**	43F	Endometriosis	Left leg, Pulmonary, Coronary, Splenic, and Renal	None	Ovarian CA	Mitral	None	Abdominal hysterectomy with bilateral salpingoopherecomy	Alive	−

### 3.2 Presenting symptoms

The most prevalent initial presenting symptoms that were neurologic in etiology included headache (n = 3) (Borowski et al., 2005, Numnum et al., 2006, Singh et al., 2007), hemiplegia (n = 3) (Orfanelli et al., 2016, Mukai et al., 1988, Kooiker et al., 1976), hemiparesis (n = 2) (Gilbert et al., 2017, Yagi et al., 2014), unsteady gait (n = 2) (Mukai et al., 1988, Singh et al., 2007), dizziness (n = 2) (Tanaka et al., 2009, Singh et al., 2007), apathy (n = 2) (Borowski et al., 2005, Lin et al., 2015), seizure (n = 1) (Kooiker et al., 1976), multiple falls (n = 1) (Singh et al., 2007), facial droop (n = 1) (Gilbert et al., 2017), unilateral spatial neglect (n = 1) (Yagi et al., 2014), bizarre behavior (n = 1) (Kooiker et al., 1976). Visual changes included blurred vision (n = 1) (Numnum et al., 2006) and diplopia (n = 1) (Singh et al., 2007). Changes in vocal capabilities included aphasia (n = 2) (Tadokoro et al., 2013), mutism (n = 1) (Lin et al., 2015), and malapropisms (n = 1) (Gilbert et al., 2017). Other common presenting symptoms include abdominal pain (n = 2) (Orfanelli et al., 2016, Aryana et al., 2006), fatigue (n = 2) (Numnum et al., 2006, Singh et al., 2007), fever (n = 2) (Aryana et al., 2006, Tanaka et al., 2009), ascites (n = 2) (Sawai et al., 2018, Makhdumi et al., 2021), nausea and vomiting (n = 1) (Aryana et al., 2006), and back pain (n = 1) (Yagi et al., 2014).

### 3.3 Past medical history

Of the 15 cases, past medical history was described in ten patients. The most common past medical history included ovarian cancer (n = 5) (Orfanelli et al., 2016, Sawai et al., 2018, Lin et al., 2015, Makhdumi et al., 2021, Yagi et al., 2014), diabetes mellitus (n = 2) (Lin et al., 2015, Singh et al., 2007), hypertension (n = 2) (Lin et al., 2015, Singh et al., 2007), dyslipidemia (n = 1) (Tadokoro et al., 2013), stroke (n = 1) (Borowski et al., 2005), myocardial infarction (n = 1) (Mukai et al., 1988), iron deficiency anemia (n = 1) (Numnum et al., 2006), and endometriosis (n = 1) (Numnum et al., 2006).

### 3.4 Diagnostic presentation

Diagnosis at presentation was provided in all cases (n = 15). The most common diagnostic presentation was a CVA at 53.3 % (n = 8) (Borowski et al., 2005, Gilbert et al., 2017, Tanaka et al., 2009, Lin et al., 2015, Kooiker et al., 1976, Singh et al., 2007, Tadokoro et al., 2013, Tadokoro et al., 2013), MI 13.3 % (n = 2) (Orfanelli et al., 2016, Mukai et al., 1988), disseminated intravascular coagulation (DIC) 6.67 % (n = 1) (Yagi et al., 2014), Trousseau’s syndrome 6.67 % (n = 1) (Sawai et al., 2018), mitral valve prolapse 6.67 % (n = 1) (Numnum et al., 2006), aortic regurgitation 6.67 % (n = 1) (Makhdumi et al., 2021) and endometriosis 6.67 % (n = 1) (Aryana et al., 2006).

### 3.5 Diagnostic work-up

Blood cultures were negative in 100 % of the cases in which they were performed (n = 13). Workup with either TTE or TEE was performed in 11 patients Table 2. Of these 11 patients, one or more valvular vegetations was identified in 100 % of patients. Two patients died before echocardiography was performed, but had autopsy findings which revealed one or more valvular vegetations in both patients (Orfanelli et al., 2016, Kooiker et al., 1976). For the other two patients, the paper confirmed vegetations were identified but did not specify the method by which valvular vegetations were identified (Tadokoro et al., 2013, Tadokoro et al., 2013). When blood work was abnormal, it most often revealed thrombocytopenia (n = 6), elevated CA-125 (n = 6), elevated D-dimer (n = 5), and abnormal inflammatory markers (n = 3).

**Table 2:** Diagnostic work-up.

Paper	Patient	Blood Culture	Echocardiogram	CT chest findings
Makhdumi et al.	48F	Negative	Mild aortic regurgitation, moderate aortic regurgitation	Small pleural effusions bilaterally
Yagi et al.	54F	Negative	Mobile vegetations on the mitral and aortic valves	Pulmonary Embolism
Gilbert et al.	58F	Negative	Two mobile echo-densities on the atrial surface of the mitral valve leaflets	Not performed
Sawai et al.	53F	Negative	Severe aortic regurgitation with vegetations	Not performed
Kooiker et al.	51F	−	Not performed − discovered on autopsy	Not performed
Numnum et al.	38F	Negative	Mild mitral valve prolapse, mild mitral regurgitation, and severe aortic regurgitation. Trileaflet aortic valve with thickened leaflets and a 10-mm mobile vegetation present on the noncoronary cusp of the aortic valve. Worsening aortic insufficiency with severe cusp retraction secondary to vegetations.	Not performed
Singh et al.	52F	Negative	7 mm vegetation on the anterior mitral leaflet	Multiple pulmonary emboli in the right lung
Borowski et al.	46F	Negative	Mild aortic regurgitation, vegetations smaller than 5 mm in diameter attached to the left coronary and noncoronary aortic cusps.	Unremarkable
Mukai et al.	40F	Negative	Cusps of the aortic valve and mitral valve leaflets were thick and uneven suggesting vegetations	Not performed
Lin et al.	60F	Negative	Severe mitral regurgitation and oscillation, with moderate-sized vegetation on the mitral valve leaflet and tricuspid valve leaflet	Not performed
Tadokoro et al.	55F	Negative	Unspecified	Unspecified
Tadokoro et al.	56F	Negative	Unspecified	Unspecified
Tanaka et al.	46F	Negative	Nodules about 10 mm in size, and mobile echo density on the atrial surface of the anterior mitral valve leaflet, associated with moderate mitral regurgitation.	Pleural effusion and ground-glass opacity was seen in the lower lobe of left lung
Orfanelli et al.	59F	−	Not performed	Not performed
Aryana et al.	43F	Negative	0.7 x 0.9 cm nodular, Mobile echodensity on the atrial surface of the anterior mitral valve leaflet associated with severe mitral regurgitation, a 0.5 by 0.6 cm nodular echodensity attached to the Eustachian valve in the right atrium; and 0.8 by 1.0 cm and 0.7 by 0.9 cm nodular mobile echodensities attached to the chordal structures in the right ventricle.	Not performed

Of the 15 patients, 12 had reported infarcts. 91.7 % (n = 11) of these patients experienced multiorgan infarct. There were 37 total organs infarcted, with the kidney comprising 29.7 % of all the infarcts (n = 11) followed by the brain 27.0 % (n = 10), spleen 27.0 % (n = 10), lung 8.1 % (n = 3), and heart 8.1 % (n = 3). All patients but one showed signs of a hypercoagulable state which included CVA (n = 8) (Borowski et al., 2005, Kooiker et al., 1976, Yagi et al., 2014, Sawai et al., 2018, Tanaka et al., 2009, Lin et al., 2015), DVT (n = 7) (Aryana et al., 2006, Sawai et al., 2018, Tanaka et al., 2009, Numnum et al., 2006, Singh et al., 2007, Tadokoro et al., 2013, Tadokoro et al., 2013), MI (n = 2) (Orfanelli et al., 2016, Mukai et al., 1988), DIC (n = 2) (Orfanelli et al., 2016, Tanaka et al., 2009), and PE (n = 1) (Yagi et al., 2014). One patient case did not show any signs of a hypercoagulable state. Makhdumi et al., 2021 .

### 3.6 Valvular Localization

As described above, all 15 patients had one or more valvular vegetations. Echocardiographic evidence of vegetation was found in 11 of the 15 patients. In comparison, autopsy evidence of vegetation was found in two patients, and two additional patients were described as having vegetations with an unspecified method of visualization. In our 15 patients, a total of 21 valves were affected. The mitral valve was the most commonly affected at 52.4 % (n = 11) (Orfanelli et al., 2016, Aryana et al., 2006, Gilbert et al., 2017, Tanaka et al., 2009, Lin et al., 2015, Mukai et al., 1988, Kooiker et al., 1976, Yagi et al., 2014, Singh et al., 2007, Tadokoro et al., 2013), followed by the aortic valve at 33.3 % (n = 7) (Borowski et al., 2005, Orfanelli et al., 2016, Sawai et al., 2018, Tanaka et al., 2009, Lin et al., 2015, Mukai et al., 1988, Kooiker et al., 1976, Yagi et al., 2014, Singh et al., 2007), and the tricuspid at 14.3 % (n = 3) (Orfanelli et al., 2016, Lin et al., 2015, Numnum et al., 2006).

### 3.7 Time to diagnosis of NBTE/Ovarian cancer

In cases with a specified timeline, ovarian cancer (n = 8) (Orfanelli et al., 2016, Aryana et al., 2006, Sawai et al., 2018, Lin et al., 2015, Makhdumi et al., 2021, Yagi et al., 2014, Tadokoro et al., 2013, Tadokoro et al., 2013). was predominantly diagnosed before NBTE (n = 6) (Borowski et al., 2005, Gilbert et al., 2017, Tanaka et al., 2009, Mukai et al., 1988, Numnum et al., 2006, Singh et al., 2007). Notably, one patient had neither ovarian cancer nor NBTE diagnosed while alive and was discovered concurrently during autopsy. Kooiker et al., 1976 .

### 3.8 Metastasis

The site of metastatic spread was described in five of the 15 patients (Borowski et al., 2005, Orfanelli et al., 2016, Gilbert et al., 2017, Sawai et al., 2018, Lin et al., 2015). The most common site of metastasis included lymph nodes 40 % (n = 2) (Borowski et al., 2005, Lin et al., 2015), liver 40 % (n = 2) (Orfanelli et al., 2016, Sawai et al., 2018), and the other ovary 20 % (n = 1) (Gilbert et al., 2017). Cancer stage was specified in nine of the 15 patients (Borowski et al., 2005, Orfanelli et al., 2016, Aryana et al., 2006, Numnum et al., 2006, Sawai et al., 2018, Tanaka et al., 2009, Lin et al., 2015). Most patients were stage IV 33.3 % (n = 3) (Borowski et al., 2005, Lin et al., 2015, Makhdumi et al., 2021), followed by stage III 33.3 % (n = 3) (Orfanelli et al., 2016, Gilbert et al., 2017, Numnum et al., 2006), stage II 11.1 % (n = 1) (Aryana et al., 2006), and stage I 22.2 % (n = 2). Sawai et al., 2018, Tanaka et al., 2009.

### 3.9 Treatment

Patients were treated with anticoagulation in 53.8 % (n = 7) (Orfanelli et al., 2016, Numnum et al., 2006, Singh et al., 2007, Tanaka et al., 2009, Lin et al., 2015) of the 13 specified cases. Of the seven cases treated with anticoagulation, four patients were treated with a single anticoagulant, including heparin infusion (n = 3) (Borowski et al., 2005, Orfanelli et al., 2016, Singh et al., 2007) and warfarin (n = 1) (Numnum et al., 2006). Two patients in this group were treated with multiple anticoagulants, including one patient treated with rivaroxaban followed by heparin (n = 1) (Sawai et al., 2018) and another treated with ticlopidine, warfarin, and heparin (n = 1) (Lin et al., 2015). One case stated that anticoagulation was given, but the paper does not specify what type(s) (Tanaka et al., 2009). Of the six cases not treated with anticoagulation, the most common rationale for not anticoagulating was intracranial hemorrhage (Aryana et al., 2006, Gilbert et al., 2017, Mukai et al., 1988, Kooiker et al., 1976, Makhdumi et al., 2021, Yagi et al., 2014). Two cases did not specify whether anticoagulation was given (Tadokoro et al., 2013, Tadokoro et al., 2013). Four patients underwent valvular surgery for NBTE (Borowski et al., 2005, Lin et al., 2015, Numnum et al., 2006, Makhdumi et al., 2021). The mortality rate of the four patients who underwent valvular surgery was 50 % (n = 2) (Borowski et al., 2005, Lin et al., 2015). Antibiotics were administered in 53.8 % (n = 7) (Borowski et al., 2005, Aryana et al., 2006, Numnum et al., 2006, Tanaka et al., 2009, Lin et al., 2015) of the 13 specified cases. In two patient cases, it was not specified whether antibiotics were administered (Tadokoro et al., 2013, Tadokoro et al., 2013). Five patients were newly initiated on chemotherapy (Orfanelli et al., 2016, Aryana et al., 2006, Gilbert et al., 2017, Numnum et al., 2006, Rahouma et al., 2023) upon diagnosis of NBTE and ovarian cancer and three patients were confirmed not to have received chemotherapy (Tanaka et al., 2009, Mukai et al., 1988, Kooiker et al., 1976). Seven cases did not specify whether chemotherapy was used or identified remote use of unspecified chemotherapy for previously diagnosed cancer (Borowski et al., 2005, Sawai et al., 2018, Lin et al., 2015, Makhdumi et al., 2021, Yagi et al., 2014, Tadokoro et al., 2013, Tadokoro et al., 2013). Additionally, all five patients who underwent chemotherapy for newly diagnosed ovarian cancer were diagnosed with NBTE before the initiation of chemotherapy (Orfanelli et al., 2016, Aryana et al., 2006, Gilbert et al., 2017, Numnum et al., 2006, Rahouma et al., 2023). Two patients (Sawai et al., 2018, Lin et al., 2015) were diagnosed with ovarian cancer remotely (years before presenting with NBTE) and did not require additional chemotherapy at presentation. Chemotherapy regimens included carboplatin and paclitaxel (n = 3) (Orfanelli et al., 2016, Gilbert et al., 2017, Singh et al., 2007) as well as paclitaxel, carboplatin, topotecan, pegylated doxorubicin, and gemcitabine (n = 1) (Numnum et al., 2006). One patient case did not specify the type of chemotherapy (Aryana et al., 2006). No VEGF inhibitors were used. Other treatments included radiation therapy (in conjunction with chemotherapy) (n = 1) (Numnum et al., 2006) and surgical resection of ovarian cancer (n = 6) (Borowski et al., 2005, Aryana et al., 2006, Gilbert et al., 2017, Sawai et al., 2018, Lin et al., 2015, Numnum et al., 2006).

### 3.10 Summarization of causes of death

The mortality rate was 75 % (n = 9) (Borowski et al., 2005, Orfanelli et al., 2016, Sawai et al., 2018, Tanaka et al., 2009, Lin et al., 2015, Mukai et al., 1988, Kooiker et al., 1976, Yagi et al., 2014, Singh et al., 2007) among the 12 patients in which mortality was described at the time of case report publication. Three cases did not report death or survival (Makhdumi et al., 2021, Tadokoro et al., 2013, Tadokoro et al., 2013). Of the nine deaths, six were most likely a direct result of NBTE (Orfanelli et al., 2016, Sawai et al., 2018, Tanaka et al., 2009, Mukai et al., 1988, Kooiker et al., 1976, Yagi et al., 2014). Causes of death among this group included DIC (n = 3) (Orfanelli et al., 2016, Mukai et al., 1988, Yagi et al., 2014), multiorgan failure (n = 1) (Sawai et al., 2018), cerebral infarction (n = 1) (Kooiker et al., 1976), and decompensation leading to death (n = 1) (Tanaka et al., 2009). Three deaths were not caused by sequelae of NBTE and include two caused by ovarian cancer (Borowski et al., 2005, Singh et al., 2007) and one by sepsis (n = 1) (Lin et al., 2015). Mortality rate stratified by valve affected was highest in the group that had aortic valvular involvement 100 % (2 of 2) (Borowski et al., 2005, Sawai et al., 2018), then multivalvular disease 80 % (4 of 5) (Orfanelli et al., 2016, Lin et al., 2015, Mukai et al., 1988, Yagi et al., 2014), and lastly mitral valve disease only 75 % (3 of 4) (Tanaka et al., 2009, Kooiker et al., 1976, Singh et al., 2007). Notably, of the three patients who were confirmed to have survived at study publication, 33.3 % (n = 1) (Numnum et al., 2006) received anticoagulation during their hospitalization. Of the nine who died, 66.7 % (n = 6) (Orfanelli et al., 2016, Singh et al., 2007, Tanaka et al., 2009, Lin et al., 2015) received anticoagulation. The sole patient who received a DOAC had expired at study conclusion (Sawai et al., 2018). Among the two patients who received warfarin, the mortality rate was 50 % (n = 1) (Lin et al., 2015) Among the four patients who received heparin, the mortality rate was 100 % (n = 4) (Borowski et al., 2005, Orfanelli et al., 2016, Sawai et al., 2018, Singh et al., 2007). Of the five patients newly initiated on chemotherapy, mortality rate was 40 % (n = 2) (Orfanelli et al., 2016, Singh et al., 2007). This is contrasted with a 100 % mortality rate (n = 3) (Sawai et al., 2018, Mukai et al., 1988, Kooiker et al., 1976) in the three patients not treated with chemotherapy. The mortality rate of the patients who underwent treatment with surgical resection of ovarian cancer was 41.7 % (n = 5) (Borowski et al., 2005, Aryana et al., 2006, Gilbert et al., 2017, Sawai et al., 2018, Numnum et al., 2006). For the one patient who was treated with radiation in conjunction with chemotherapy, the mortality rate was 0 % (n = 1) (Numnum et al., 2006).

## 4 Discussion

NBTE, also known as marantic endocarditis, is a rare condition frequently associated with advanced malignancies and autoimmune disorders. Importantly, it is characterized by the presence of sterile vegetations on heart valves composed of fibrin and platelet aggregates without inflammatory or bacterial cells (Asopa et al., 2007). NBTE usually remains asymptomatic until patients present with a sentinel event, usually from systemic embolic events, the most common of which include cerebrovascular accidents (CVA), organ infarcts, and peripheral embolisms (Dafer, 2021). Less commonly, cardiac issues such as dyspnea, heart failure, and valvular dysfunction also occur. Due to the indolent nature of NBTE, the underlying etiology can be difficult to decipher. Currently, published literature on NBTE is largely limited to case reports, and includes involvement with lung, breast, pancreatic, and other malignancies. We reviewed the literature to better understand the clinical course and outcomes in NBTE associated with ovarian cancer.

Our analysis described 15 female patients with an average age of 50.6 years, in-line with a long-term study of cancer related NBTE from Cleveland Clinic with a female-predominant population averaging 54 years of age (Zmaili et al., 2021). Presenting symptoms included headaches, gait instabilities, and neurological symptoms such as vision changes, speech difficulties, and seizures. In addition to the above sequelae of thromboembolic events, patients also presented with signs and symptoms concerning for malignancy including abdominal pain, fatigue, fever, ascites, and nausea/vomiting. Due to the variety of presenting symptoms, the diagnosis of NBTE can be challenging. We found that the most common initial diagnosis for patients included CVA and myocardial infarction (MI), alluding to the thromboembolic nature of NBTE.

The most common cause of a CVA is an ischemic stroke from hypoperfusion or thromboembolic events (AbuRahma et al., 2022). In our study, we found that prior medical history was mentioned in 10 patients and included ovarian cancer, diabetes mellitus (DM), hypertension (HTN), stroke, and MI. Aside from ovarian cancer, which was diagnosed in half the patients with a mentioned medical history, many patients did not demonstrate prothrombotic conditions to explain thromboembolic events. As such, unexplained thromboembolic events can pose significant diagnostic challenges and should prompt a further workup.

Our study found that ovarian cancer was diagnosed before NBTE in most patients. Ovarian cancer has a variable prognosis depending on the stage at diagnosis. Early-stage disease has a favorable prognosis with five-year survival rates as high as 90 % (Colombo et al., 2006). Conversely, advanced-stage ovarian cancer has a much poorer prognosis, with five-year survival rates of around 25 % (Jayson et al., 2014). Cancer staging was specified in half of our cases, with most diagnoses occurring at advanced stages resulting in a generally poor overall prognosis. Notably, most NBTE cases were diagnosed after an ovarian cancer diagnosis, leading to the association with advanced malignancies (Zadok et al., 2022). It is possible that patients diagnosed with advanced malignancies receive a larger diagnostic workup, leading to the discovery of NBTE.

NBTE is diagnosed clinically after excluding microbiological causes and echocardiographic findings (Tonutti et al., 2023). Definitive confirmation of NBTE is provided with histopathological examination of the excised cardiac valve, however, it is typically not performed due to the invasive nature and risks of the procedure. Patients in the studied cases had either a TTE or a TEE with findings of one or more valvular lesions. Most patients had vegetations most commonly affecting the mitral valve followed by the aortic valve. Patients also had multi-valvular disease with various combinations of valves, however, all with left-sided involvement. Left-sided valves are more prone to vegetations due to higher pressure gradients and turbulent flow leading to endothelial damage and calcification (Bentata, 2017). Peripheral embolic events are also common NBTE and as such our data reflects this. Many of the patients in our analysis showed multi-organ infarcts with the most common being the kidney, brain, and spleen. A similar distribution is seen in infective endocarditis likely due to the rich blood supply within these organs as well as the left-sided valvular predisposition leading to systemic access (Baddour et al., 2005).

Treatment for NBTE consists of anticoagulation and addressing the underlying malignancy. The most cited anticoagulation in the literature is warfarin, however, the use of direct oral anticoagulants (DOACs) has also been documented (Slivka et al., 2021). We found that only about half of the patients in our analysis were treated with anticoagulation, the most common of which was unfractionated heparin. The most frequently documented contraindication to anticoagulation was intracranial hemorrhage, but some cases did not clearly define a reason for foregoing anticoagulation. Although a fundamental aspect of treatment, there is no standardization on timing of anticoagulation or agent of choice. Unfractionated heparin was likely the most common due to its ease of cessation if clinically warranted. Interestingly, antibiotics were also administered in some cases, possibly due to early initiation prior to a diagnosis of NBTE or due to an inability to rule out infective endocarditis. Chemotherapy and radiation therapy was also pursued in several cases, likely owing to the good response rate in ovarian cancer (Slivka et al., 2021).

At the time of publication, the mortality rate was high at 75 % in our subset of patients. Although a majority of patients were diagnosed with advanced stage ovarian cancer, it was not the primary cause of death in most patients. NBTE-related complications contributed to the high mortality with left-sided valvular lesions causing the greatest mortality. Despite anticoagulation therapy being used in over half of the cases, there was no clear reduction in mortality. This extends to patients who were treated with DOACs as well as those treated with warfarin. Although a population-based cohort study found that warfarin has been shown to have higher overall survival rates compared to DOACs in cancer-associated VTE, results are inconsistent in the literature and our results are not powered to draw such conclusions (Khan et al., 2022). More studies are needed with larger patient populations to examine the differences in mortality between vitamin K antagonists and DOACs in ovarian cancer-related NBTE. Most epithelial ovarian cancer is responsive to chemotherapy and as such our results demonstrate this with patients having decreased mortality following chemotherapy treatment. It is possible that the cohort that received chemotherapy might have been healthier with less comorbidities than those who did not. Despite toxic side effects, it appears that targeting fast-dividing cancer cells and potentially reducing tumor burden is beneficial in patients with NBTE (el-Shami et al., 2007). Sex appears to play a smaller role in overall mortality, as most patients with epithelial ovarian cancer are post-menopausal and therefore have lower estrogen levels, reducing the potential hormonal contribution to hypercoagulability compared to men. While most reported cases of NBTE in ovarian cancer involved serous adenocarcinoma, this may reflect the predominance of this subtype rather than a true increased risk. Interestingly, studies on venous thromboembolism have identified certain histologic subtypes, such as clear cell carcinoma, as having a higher risk of thromboembolic events compared to other forms of epithelial ovarian cancer (Weeks et al., 2020, 2020.). However, additional studies are needed to better understand the relationship between NBTE and specific ovarian cancer subtypes.

There are several limitations to our study. Due to the scarce nature of ovarian cancer related NBTE, our literature search was limited to case reports. As such, our results cannot be extrapolated to the general population nor have the power to draw definite conclusions. Physicians should recognize NBTE on the differential diagnosis when addressing patients presenting with neurological sequela and high-risk features for underlying malignancy. Our results have shown that mortality rates are high with NBTE in ovarian cancer. Although it can present with debilitating symptoms and have long-term complications, there is no standard of care for treatment. We highlight an area with little research and high mortality for patients suffering from ovarian cancer. Further large-scale studies need to be performed to establish more robust data on the diagnosis, timing of anticoagulation and agent of choice, and outcomes of NBTE in ovarian cancer.

## 5 Conclusions

NBTE is a dangerous complication of malignancies and other hypercoagulable/inflammatory states which can lead to thromboembolic phenomena. The literature has shown a compelling association specifically between ovarian cancer and NBTE. Prompt recognition and treatment of NBTE in ovarian cancer can be key to patient survival. Clinicians should have high clinical suspicion for NBTE in ovarian cancer with any evidence of thromboembolic disease. Given the rarity of NBTE in ovarian cancer, further collaborative efforts are needed to help identify at-risk patients and to develop more specific treatment guidelines.

Funding.

This work was not financially supported by any agencies, individuals, or other sources.

Author contributions.

The authors confirm their contributions to the paper:

● Conceptualization and Methodology: Hussain, Fahad; Paracha, Awais
● Formal Analysis and Investigation: Hussain, Fahad; Paracha, Awais; Huba, Mahalia; Guntaka, Saimanoj; John, Veena
● Original Draft: Huba, Mahalia; Hussain, Fahad; Guntaka, Saimanoj; Paracha, Awais; Sathe, Pranav; Parikh, Bhavya; Noyelle, Margot; Durrani, Umar; Patel, Himanshu; John, Veena

All authors reviewed the results and approved the final version of the manuscript.

## CRediT authorship contribution statement

**Mahalia Huba:** Writing – review & editing, Writing – original draft, Data curation. **Fahad Hussain:** Writing – review & editing, Writing – original draft, Conceptualization. **Saimanoj Guntaka:** Writing – review & editing, Writing – original draft. **Awais Paracha:** Writing – review & editing, Writing – original draft, Conceptualization. **Pranav Sathe:** Writing – review & editing, Writing – original draft. **Bhavya Parikh:** Writing – review & editing, Writing – original draft. **Margot Noyelle:** Writing – review & editing, Writing – original draft. **Umar Durrani:** Writing – review & editing, Writing – original draft. **Himanshu Patel:** Writing – review & editing, Writing – original draft. **Veena John:** Writing – review & editing, Formal analysis, Conceptualization.

## Declaration of competing interest

The authors declare that they have no known competing financial interests or personal relationships that could have appeared to influence the work reported in this paper.
